# The Potential of Microalgae–Bacteria Consortia to Restore Degraded Soils

**DOI:** 10.3390/biology12050693

**Published:** 2023-05-09

**Authors:** Lina M. Gonzalez-Gonzalez, Luz E. de-Bashan

**Affiliations:** 1 The Bashan Institute of Science, 1730 Post Oak Ct, Auburn, AL 36830, USA; 2 Department of Entomology and Plant Pathology, Auburn University, 209 Life Sciences Building, Auburn, AL 36849, USA; 3 Environmental Microbiology Group, Northwestern Center for Biological Research (CIBNOR), Avenida IPN 195, La Paz 23096, Mexico

**Keywords:** microalgae, cyanobacteria, plant growth-promoting bacteria, degraded soils, bioremediation, restoration

## Abstract

**Simple Summary:**

Beneficial microorganisms, such as microalgae and bacteria, have a strong ability to restore health and fertility in degraded soils. However, the use of these microorganisms interacting in a mixed consortium has yet to be well explored. Furthermore, most of the current knowledge on the effects of these microorganisms on soil fertility derives from studies focused on the potential of either of these groups as biofertilizers; thus, more information on their real impact on degraded soils is required. This mini-review addresses the current knowledge on using a consortium of microalgae and bacteria for this purpose.

**Abstract:**

Soil restoration is one of the biggest challenges of this century. Besides the negative impacts of climate change, the current increase in food demands has put severe pressure on soil resources, resulting in a significant area of degraded land worldwide. However, beneficial microorganisms, such as microalgae and plant growth-promoting bacteria, have an outstanding ability to restore soil health and fertility. In this mini-review, we summarize state-of-the-art knowledge on these microorganisms as amendments that are used to restore degraded and contaminated soils. Furthermore, the potential of microbial consortia to maximize beneficial effects on soil health and boost the production of plant-growth-promoting compounds within a mutualistic interaction is discussed.

## 1. Introduction

The ability to meet the continuous increase in food demands as a consequence of population growth is one of the biggest challenges of this century. Climate change and freshwater limitations are realities that we must consider when developing sustainable agricultural systems [1]. The current increase in food demands has put severe pressure on soil resources, resulting in significant areas of degraded soil worldwide due to intensive and poor agricultural land management [2,3]. Similarly, industrial activities, modern agricultural practices, improper waste disposal, and accidental spills of hazardous substances result in soil contamination [4,5]. Moreover, climate change alters fire regimes, significantly affecting soils and ecosystems [6]. Sadly, more than 33% of global land is degraded, and this percentage will grow if no actions are taken to prevent and reverse the degradation [7].

One of the first steps in tackling the increasing food demands is restoring degraded soils (i.e., degraded farmlands, contaminated soils, and post-fire ecosystems). The use of organic amendments, such as microalgae, especially cyanobacteria, is an increasing field of study that aims to restore soil health and fertility [6,8,9]. These microorganisms have the capability to restore soil structure and aggregate stability by releasing exopolysaccharides and forming soil aggregates, providing O_2_ to the subsurface, solubilizing and mobilizing macro- and micronutrients, mineralizing simpler organics, and serving as a source of organic matter and nutrients [10,11]. Additionally, the fertilizer potential of these microorganisms is well documented. Microalgae, including cyanobacteria, contain some plant-growth-promoting substances, such as phytohormones (auxins, cytokinins, abscisic acid, ethylene, and gibberellins), amino acids, vitamins, polyamines, betaines, protein hydrolysates, and polysaccharides, which can be used as biostimulants [12]. Furthermore, microalgae extracts have a recognized potential to improve soil physical and biological properties by acting as organic slow-release fertilizers that can return nutrients (carbon and macro-elements) and ensure the efficient use of resources [13].

Plant-growth-promoting bacteria (PGPB) are bacterial strains isolated from diverse environments with the potential to positively influence the growth and yield of diverse plants, mostly of agricultural importance [14]. In recent years, the use of PGPB has been explored beyond agricultural practices toward environmental applications, such as the restoration of eroded desert soils [15,16]. Inoculation with PGPB affects the growth and metabolism of plants through a multitude of mechanisms, such as nitrogen fixation, phosphorus, iron solubilization, the production of indoleacetic acid (IAA) and other plant hormones, the production of signal molecules, and the mitigation of environmental stresses [17,18]. Several comprehensive and critical reviews describing the operational mechanisms of PGPB have been published [19,20]. Consequently, a general discussion of the likely mechanisms for promoting plant growth is not described in this review.

The use of microalgae and bacteria for the bioremediation of contaminated soils has also been explored, with promising results. Contaminants, such as heavy metals, pesticides, and hydrocarbons, can have adverse effects on soil quality and ecosystem health [4,5]. These microorganisms offer an environmentally friendly and cost-effective solution for reducing soil pollution by breaking down pollutants and transforming them into less harmful forms [21,22]. The use of these natural agents in soil remediation strategies has the potential to reduce reliance on chemical-based treatments and to promote the development of more sustainable and eco-friendly agricultural practices.

Despite their proven fertilizer and bioremediation potentials, the capacity of these microorganisms to restore degraded soils needs to be better explored. Most studies have focused on the positive effects of microalgae and PGPB on plant growth, and all aspects regarding soil health and fertility still need to be assessed. Moreover, the use of microalgae and bacteria consortia for soil restorations has been poorly studied, even though their synergistic interaction can significantly boost their positive impact on soil fertility. Microalgae and bacteria exhibit positive interactions through substrate exchange, cell-to-cell communication via small signaling molecules, and horizontal gene transfer, conferring adaptive advantages to environmental stressors [23,24].

This mini-review discusses the current knowledge of microalgae (including cyanobacteria) and PGPB as promoters of soil recovery. Furthermore, we explore the use of artificial microalgae–bacteria consortia as a promising organic amendment to restore marginal and degraded soils.

## 2. Climate Change and Soil Degradation

Soil degradation is an increasing threat of climate change that entails the loss of biodiversity and ecosystem services [25,26]. Soils provide valuable ecosystem services, such as sustainable plant production, the control of water quality, the control of biological pests and diseases, the filtering of nutrients and contaminants, carbon storage, greenhouse gas regulation, waste detoxification and recycling, and flood and climate change mitigation, among others [27,28,29,30]. Besides the obvious negative environmental impacts of dropping such services, there is also an important economic cost to society [31,32]. Additionally, when the soil becomes degraded, it loses its ability to support a diverse range of microorganisms, leading to a decline in soil microbial diversity, complexity, and functionality [33,34,35]. This, in turn, can have far-reaching consequences for the health of ecosystems and for human health and well-being. The loss of soil microbial diversity can disrupt soil ecosystem functioning by altering nutrient cycling, decreasing soil fertility, and increasing the risk of soil erosion and pollution [36,37,38]. Therefore, soil microbial biomass is widely regarded as an indicator of soil fertility and ecosystem productivity, and is a fundamental characteristic that plays a key role in soil restoration [39].

Since population growth is associated with ever-increasing food and water consumption, one of the biggest challenges that humanity is facing in the current climate change scenario is the increased degradation of arable land around the world, which is linked to food and nutritional insecurity [40,41]. Furthermore, exposure to soil contaminants and the adverse effects of their toxicity will be impacted by climate change. Changes in environmental conditions might modify the mobilization, transfer, behavior, concentration, deposition, and fate of pollutants [42,43]. Additionally, increased temperatures and salinity might increase the chemical toxicity of certain soil contaminants by altering the contaminant’s biotransformation into more bioactive metabolites [43].

There is a real threat of human displacement during the next decade because of land degradation, which encroaches on over one-third of global land [44]. In a comprehensive review, Prăvălie [45] reported several pressures around the globe that result in land degradation, such as coastal erosion, biological invasions, aridity, land erosion by water or wind, land subsidence, landslides, permafrost thawing, soil biodiversity loss, soil compaction, soil organic carbon loss, waterlogging, salinization, soil sealing, and vegetation degradation. Aridity, land erosion by water, salinization, soil organic carbon loss, and vegetation degradation were described as major degradation pathways. Land degradation leads to the transformation of grasslands into hyper-arid and desert environments, reaching up to 41.3% of the global land’s surface [46]. Furthermore, globally, the main pressures driving arable land degradation are aridity and soil erosion, which affect 40% and 20% of arable land, respectively [47].

The world’s degraded land mapped by Gibbs and Salmon [2] provides a general idea of the complex situation in many parts of the world. Ironically, global warming not only triggers land degradation but also renders degraded land less resilient and highly vulnerable to climate change [41]. Furthermore, since agricultural production is limited by the increasing scarcity and diminishing quality of land and water resources [48], the required increment in production must align with sustainable agricultural techniques and the conscious use of water resources. Avoiding agricultural expansion at the expense of forestland is mandatory, which only intensifies the environmental threat [2,47]. In this sense, restoration plans should focus on recuperating native ecosystems rather than expanding agricultural land in areas that were once forests or savannas [2].

## 3. Use of PGPB and Microalgae for Restoration of Degraded Soil

PGPB are beneficial in harsh and limiting environments because of their role in alleviating stress in plants, making them excellent candidates to assist revegetation of eroded zones. For instance, PGPB can help plants tolerate drought stress by improving their water and nutrient uptake [49]. There are several examples of soil restoration with plants inoculated with PGPB; a severely eroded land in the southern Sonoran Desert was restored using native leguminous trees and the giant cardon cactus inoculated with two PGPB (*Azospirillum brasilense* and *Bacillus pumilus*), native arbuscular mycorrhizal fungi, and small quantities of compost [16,50]. Over a decade later, highly eroded land, destroyed for 25 years with almost no topsoil and extremely low mineral quantities to support plant growth, was successfully recovered. Likewise, the outdoor nursery cultivation of mesquite tree transplants was evaluated as a way to restore arid zones [51]. The study showed that inoculating the seedlings with PGPB—*A. brasilense* immobilized in dry alginate microbeads—resulted in the enhancement of all growth parameters of the plants, including biomass, aerial volume, root system, and chlorophyll pigments. Ramachandran and Radhapriya [52] explored a similar approach in a highly degraded forest in the Nanmangalam Reserve Forest in the Eastern Ghats of India. The authors planted 12 native tree species inoculated with a consortium of five native types of PGPB, small amounts of compost, and chemical fertilizer. The results of an experiment that lasted almost three years revealed that the PGPB consortium enhanced plant biomass in all the native plants and improved soil quality in the degraded forest. Schoebitz et al. [53] evaluated the combined effect of *A. brasilense*, *Pantoea dispersa*, and an organic olive residue immobilized in clay in the revegetation of semiarid land. The study revealed that PGPB improved soil properties by increasing phosphorus and potassium content availability by up to 100% and 70%, respectively. The inoculant also increased the total carbon and microbial biomass carbon content and enzyme activities, such as dehydrogenase, urease, and protease.

High soil salinity is another undesirable feature that reduces soil fertility [54]. However, salt-tolerant PGPB can significantly enhance salt tolerance in plants through several mechanisms, such as the adjustment of osmosis, protection from free radicals, the excretion of phytohormones that enhance growth parameters, and the release of extracellular polymeric substances (EPSs) that bind with Na^+^ cations, decreasing its bioavailability for plant uptake [55,56,57]. For instance, the PGPB *Bacillus pumilus* strain JPVS11, improved the growth performance of rice (*Oryza sativa* L.), which was negatively impacted by high soil salinity [58]. The study also revealed a significant improvement in soil enzyme activities of up to 56%, 46%, 48%, and 56% in alkaline phosphatase, acid phosphatase, urease, and β-glucosidase, respectively. Likewise, Hafez et al. [59] evaluated the potential of PGPB—*Azospirillum brasilense*—to restore saline–sodic soils. Following the inoculation of the strain with eco-friendly organic wastes for 150 days, the authors reported that soil fertility was enhanced with increases in soil organic carbon, dehydrogenase, urease enzymes, micronutrients (Fe, Zn, Mn, Cu, and B), and macronutrients (N, P, and K).

The soil restoration potential of microalgae, especially cyanobacteria, has been studied far more extensively because of the protagonist-like role they play in biological soil crust or biocrust. Biocrust corresponds to a cohesive and thin horizontal ground cover composed of photosynthetic organisms, such as lichens, bryophytes, and microalgae, and their associated bacteria, archaea, and fungi, which are of uttermost importance in stabilizing the soil against erosion [6,60]. Cyanobacteria, i.e., the first colonizers, stabilize the topmost layers and facilitate the formation of the soil crust with other microalgae groups and bacteria [61]. This is particularly important in arid or semiarid lands, desertified soils, and soils affected by fire, where cyanobacteria can be a suitable soil amendment that increases nutrient availability and promotes plant growth [61]. Additionally, microalgae act as biostimulants, affecting soil biological activity by enhancing enzymatic activity [62].

Wang et al. [63] reported the suitability of an artificial consortium composed of the cyanobacterial species *Microcoleus vaginatus* and *Scytonema javanicum* to recover the biological soil crust of degraded soil in a desert area in Inner Mongolia. After cyanobacterial inoculation, the authors reported a significant increase in total nitrogen, organic carbon, total salt, calcium carbonate, and electrical conductivity. The inoculation of this consortium with the plant *Salix mongolica* was later evaluated by Lan et al. [64]. Cyanobacteria inoculation quickly formed a biocrust and gradually gave rise to the moss crust, helping vascular plants to regenerate. A similar study reported on the inoculation of a cyanobacterial consortium with the species *Anabaena doliolum*, *Cylindrospermum sphaerica*, and *Nostoc calcicole* in a semiarid clay–loam soil, improved carbon and nitrogen mineralization, increased water-holding capacity, and enhanced hydraulic conductivity. Additionally, in response to the cyanobacterial biofertilizer, pear millet and wheat crops showed an increase in their growth and yield [65]. Another artificial consortium co-formed by the filamentous cyanobacteria *Microcoleus vaginatus*, *Phormidium tenue*, *Scytonema javanicum*, *Nostoc* spp., and the chlorophycea *Desmococcus olivaceus* efficiently assisted in the stabilization of fine sands, helping to control erosion in aeolian sandy soil in the south-eastern region of the Tenger Desert [66]. Similarly, Issa et al. [67] evaluated the effect of the cyanobacteria *Nostoc* spp. on the structural stability of poorly aggregated tropical soil from the Eastern Cape Province of South Africa. Cyanobacterial inoculation increased the resistance of soil aggregates to break down, enhancing soil stability two to four times over the control after six weeks of inoculation. Another study reported the potential of the acid-tolerant microalgae species *Desmodesmus* spp. and *Heterochlorella* spp., alone, or in combination, to improve soil health and fertility. The inoculation of strains in two acid soils (Kurosol and Podosol) collected from Queensland, Australia, resulted in the development of algal soil crust. Additionally, the authors reported an increase in the release of exopolysaccharides (more than 200%) which facilitate soil stability, an increase in carbon content (up to a 57%), an increase in dehydrogenase activity (more than 500%), and an increased production of indolacetic acid (between 200 and 500%) [68]. Furthermore, the algalization of acid soils with these species enhanced the richness of ecologically important soil bacteria, such as rhizobacteria and diazotrophs [69]. Muñoz-Rojas et al. [70] also evaluated the potential of a cyanobacteria consortium with *Nostoc commune*, *Tolypothrix distorta*, and *Scytonema hyalinum* to restore mine soil and reported that up to 40% of the soil surface was covered by biocrust after 90 days, as well as a significant increase in soil organic carbon and the promotion of C sequestration.

All of these studies have revealed that the use of PGPB and microalgae, particularly cyanobacteria, is an effective approach in restoring degraded soils, increasing soil fertility, stabilizing the soil against erosion, and promoting plant growth in arid and semiarid regions. Because the inoculation of PGPB and microalgae can be a sustainable and eco-friendly strategy for soil restoration programs, the use of these microorganisms in consortia has also been explored to further enhance the positive effects of these beneficial microorganisms.

## 4. Microalgae–Bacteria Consortia as Inoculants to Restore Degraded Soils

Microbial consortia have several advantages over individual species, such as strength to environmental fluctuations, the ability to survive nutrient starvation periods by sharing metabolites, and resistance to invasion by other species [71]. The co-inoculation of different species of microalgae or bacteria in microbial consortia has shown the enhanced positive effects that each species alone has on soil fertility. The use of these microbial consortia, especially cyanobacteria species, for the restoration of degraded soils leads to significant enhancements in several traits of soil fertility (Table 1).

Furthermore, a mutualistic consortium between microalgae and bacteria can boost their metabolism [24]. Mutualism is a positive interaction between organisms of two different species in which each benefits from, and is based on, the exchange of resources and services. A mutualistic microalgae–bacteria consortium is based on the exchange of metabolites; the best-described mechanism of bacterial improvement in the growth of microalgae is supplementation with CO_2_, whereby the growth of microalgae in an environment with reduced O_2_ tension and enriched CO_2_ will increase the primary metabolism and promote an increase in their populations [72]. In return, the bacterial growth can be stimulated by the uptake of extracellular polymeric substances (EPSs) released by the microalgae, including organic carbon (myo-inositol or lactate), proteins, and amino acids (such as tryptophan) [73,74].

Alternatively, a microalgae–bacteria mutualism is established by exchanging photosynthates produced by the microalgae and vitamins produced by the bacteria, which are needed as co-factors for enzymes in key metabolic pathways in the microalgae. For example, many microalgae are auxotrophs for vitamin B12 (cobalamin) and vitamin B1 (thiamine). Because vitamin B12 is only produced by prokaryotes, it becomes the source of vitamins for the microalgae. Palacios et al. [75] reported that the production of vitamin B2 (riboflavin) and its degradation compound, lumichrome, using *Azospirillum brasilense* positively affected the microalga *Chlorella sorokiniana*. Furthermore, growth-promoting factors produced by bacteria, such as phytohormones IAA, gibberellins [76,77,78], or organic nitrogen compounds (such as uracil) [74], have been proven to have positive effects on microalgae.

**Figure 1:**
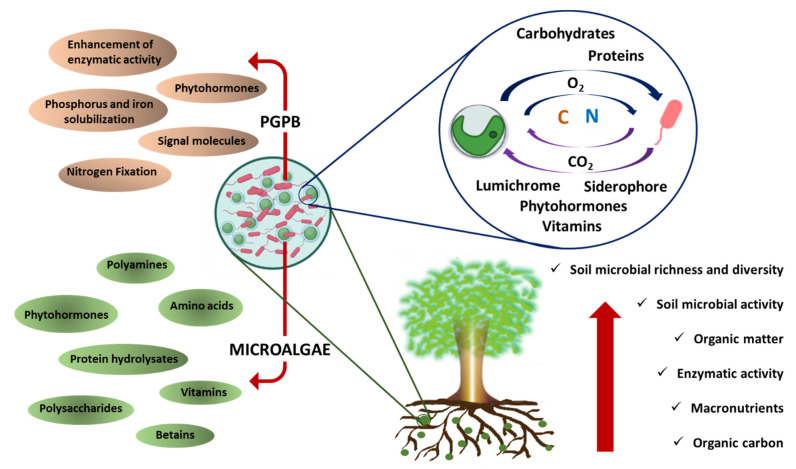
Schematic diagram of the biostimulant activity of a microalgae–bacteria consortium to restore degraded soil.

This significant enhancement in the bioactivity of both partners may lead to the enhanced capacity of microorganisms as agents used for soil restoration, as proposed in Figure 1. However, most reports of mutualism have focused on eukaryotic microalga and bacteria, and a limited number of reports found in the literature on the use of these consortia for enhancing soil characteristics mostly present the combined use of cyanobacteria and bacteria (Table 2). For example, the co-cultivation of the cyanobacteria *Anabaena variabilis* and *Nostoc calsicola* with the green microalgae *Chlorella vulgaris* and the nitrogen-fixing bacteria *Azotobacter* spp. in two different consortia (each with one cyanobacterium species) revealed the improved growth of microorganisms with the bacteria, suggesting an increase in the nitrogen-fixing activity of the consortia, which might lead to an enhancement in soil fertility [79]. In another study, Swarnalakshmi et al. [80] evaluated novel biofilm preparations using cyanobacterium *Anabaena torulosa* as a matrix for diazotrophic and phosphate-solubilizing bacteria. The authors assessed the fertilizer potential of biofilms on wheat crops and reported a significant increase in the available nitrogen, even after 14 weeks of inoculation. Similarly, the inoculation of *A. torulosa*–*Azotobacter chroococcum* biofilm on leguminous crops resulted in increases of 80% and 24% of available nitrogen and phosphorous, respectively. In the same study, *A. torulosa*–*Bradyrhizobium* spp. biofilm increased N_2_ fixation, N mobilization, and soil C sequestration [81].

Significant enhancements in microbiological and enzymatic activities have also been described following the inoculation of microbial consortia. Manjunath et al. [82] reported that the inoculation of microbial consortia (a mix of proteobacterial and cyanobacterial strains) in wheat crops enhanced dehydrogenase activity and soil microbial activity. Similarly, the inoculation of *Anabaena torulosa* in consortium with either *Rhizobium* spp. or *Pseudomonas fluorescens* in leguminous crops (chickpea, pea, and lentils) led to an increase in soil polysaccharides, dehydrogenase and nitrogenase activity, soil carbon, and available P [87]. Bidyarani et al. [88] evaluated the same cyanobacterial species in consortium with *Mesorhizobium cicero* inoculated in chickpea crops. The authors reported a significant increase in the soil available N and P, N_2_ fixation, and dehydrogenase activity. Another study of *Anabaena* sp. in consortium with the bacteria *Providencia* spp. revealed that the consortium inoculated in wheat seeds produced significant increases in alkaline phosphatase and dehydrogenase activities and microbial biomass carbon in the soil [86].

Microalgae–bacteria inoculants can also help to control soil erosion induced by rainfall. For example, the direct inoculation of selected strains of cyanobacteria (*Lyngbya* spp., *Nostoc* spp., and *Oscillatoria* spp.), and bacteria (*Azotobacter* spp. and *Bacillus subtilis*) in soil collected from an area highly susceptible to erosion effectively increased soil stability, leading to a 99% soil loss reduction rate [85].

In a few reports on the consortia between eukaryotic microalgae and bacteria, Trejo et al. [89] proved the efficiency of *Chlorella sorokiniana* and *Azospirillum brasilense* debris as an amendment for infertile soils with low levels of organic matter. After wastewater treatment, the inoculation of dried alginate beads containing *C. sorokiniana* and *A. brasilense* significantly increased the organic matter, organic carbon, and microbial carbon of eroded infertile desert soil. Later, Lopez et al. [84] revealed a significant increase in soil microbial richness and diversity following the inoculation of the same microbial consortium. Additionally, the growth of sorghum in the amended soil was greater, and the deep colonization of the root surface using *A. brasilense* was observed.

In summary, co-inoculating different species of microalgae and bacteria has been shown to enhance the positive effects of each species on soil fertility. Additionally, the mutualistic consortium between microalgae and bacteria can boost their metabolisms through the exchange of metabolites, photosynthates, and vitamins, thus enhancing the capacity of microorganisms as agents for soil restoration.

## 5. Microalgae and Bacteria for Bioremediation

Besides the natural phenomena inducing soil degradation, several contaminants, such as heavy metals, pesticides, and hydrocarbons in petroleum products, can have detrimental effects on soil quality and ecosystem health. Heavy metals, such as lead, arsenic, and cadmium, can accumulate in soil and affect plant growth and microbial activity, leading to reduced soil fertility and crop yield [90]. Similarly, polycyclic aromatic hydrocarbons (PAHs) from petroleum products can contaminate soil and cause toxic effects on soil organisms, impairing their ability to decompose organic matter and carry out nutrient cycling [91,92]. Pesticides can also have toxic effects on soil biota, including beneficial microbes and insects, leading to reduced soil biodiversity and enzymatic activity. This can result in biological and physicochemical transformations that negatively impact ecosystem function and crop productivity [93]. These contaminants can persist in the soil for long periods of time, not only affecting the immediate ecosystem but also potentially contaminating groundwater and nearby water bodies. Bioremediation with PGPB and microalgae has also been explored for the removal of these contaminants, with promising results. Microalgae and bacteria can play a crucial role in reducing contaminants from soils by breaking down pollutants and transforming them into less harmful forms [21,22]. This process helps to reduce the overall toxicity of the soil and prevents further contamination.

PGPB in phytoremediation have shown great potential by enhancing plant growth and biomass, especially under stressed conditions, resulting in faster and more efficient processes [94]. For example, Silambarasan et al. [95] reported an enhancement in the phytoremediation efficiency of *Helianthus annus* with the inoculation of *Pseudomonas citronellolis* strain SLP6. The Cu- and salinity-tolerant bacterial strains efficiently assisted in the phytostabilization of Cu-contaminated saline soils by enhancing plant growth and the Cu accumulation potential. Similarly, Rajkumar and Freitas [96] evaluated the potential of the PGPB *Pseudomonas* spp. and *Pseudomonas jessenii* as growth-promoting bioinoculants for plants and metal sequestration in soil contaminated with Ni, Cu, and Zn. The inoculation of *Ricinus communis* with PGPB increased the efficiency of phytoextraction by increasing metal solubilization in contaminated soil. Additionally, the biosorption and bioaccumulation capacities of the PGPB reduced the phytotoxic effects of the metals. In another study, the PGPB *Bacillus altitudinis* strain KP-14 successfully assisted in the phytostabilization potential of *Miscanthus x giganteus* in aged soil contaminated with several trace elements [97]. Similarly, Franchi et al. [98] reported that the combined effect of thiosulfate and a bacterial consortium increased the phytoaccumulation efficacy of Brassica juncea by up to 85% for arsenic and 45% for mercury in contaminated soil from a disused industrial area in northern Italy.

The use of PGPB in assisting in the bioremediation of pesticide residues in agricultural soils has also been explored, with promising results [99]. For instance, the potential of *Bacillus aryabhattai* to mitigate paraquat residues in drought soil was evaluated in pot experiments with cowpea seeds. The authors reported that the strain remediated the paraquat-contaminated soil and significantly improved the growth of cowpea compared to soil without bacterial inoculation [100]. Another study revealed that several species of the PGPB genus *Bacillus* were able to degrade pesticides, including acibenzolar-S-methyl, metribuzin, napropamide, propamocarb hydrochloride, and thiamethoxam, within a 72 h incubation period [101]. Similarly, the PGPB *Pseudomonas rhizophila* S211 was identified as a promising strain for the bioremediation of soils contaminated with pentachlorophenol [102], while the bacterial strains *Acinetobacter calcoaceticus* MCm5, *Brevibacillus parabrevis* FCm9, and *Sphingomonas* spp. were highly efficient in degrading cypermethrin and other pyrethroids, such as RCm6. These strains also exhibited plant-growth-promoting traits, such as phosphate solubilization and indole acetic acid and ammonia production [103].

Environmental pollution from petroleum spills is another problematic issue when restoring soil quality, and PGPB has exhibited great potential in their degradation. For example, the inoculation of PGPB increased the phytoremediation potential of *Scirpus triqueter* growing in a co-contaminated soil with Ni and pyrene, increasing the bioavailability of Ni and promoting the degradation of pyrene [104]. Similarly, Sampaio et al. [105] evaluated the biodegradation of PAHs in a diesel oil-contaminated mangrove at the Paraguassu River in Brazil. The authors monitored the ability of *Rhizophora mangle* L. to degrade PAHs in contaminated soil inoculated with PGPB *Bacillus* spp. and *Pseudomonas aeruginosa* and reported a removal rate of up to 80%. The interaction between *R. mangle* and the bacterial strains revealed the potential of the bacteria to assist in the phytoremediation of soils contaminated with diesel oil.

Furthermore, numerous studies have shown the potential of microalgae to reduce contaminant levels in soil. For example, Iliev et al. [106] evaluated the seed germination and plant growth of *Tribulus terrestris* following inoculation with a mixed algal suspension (mainly *Scenedesmus* spp. and *Nostoc* spp.) in soil contaminated with a mineral oil spill. The authors found a similar percentage of germination of seeds grown in non-polluted conditions and those grown in contaminated soil after watering with the mixed algal suspension, which suggests an efficient removal of the pollutant. Similarly, two species of green algae and five cyanobacteria were evaluated as inoculants of *Oryza sativa* in arsenic (As)-contaminated paddy soils. The inoculation, especially of the cyanobacteria *Anabaena azotica*, improved soil nutrient bioavailability, greatly enhanced rice growth, and reduced As translocation from roots to rice grains [107]. The removal of up to 78% and 48% of fluoranthene and pyrene, respectively, in contaminated soil was reported by Lei et al. [108] with the inoculation of four different microalgae species (*Chlorella vulgaris*, *Scenedesmus platydiscus*, *Scenedesmus quadricauda*, and *Selenastrum capricornutum*). The authors found that the removal rate was species-specific and toxicant-dependent. In another study, Decesaro et al. [109] compared the degradation potential of diesel and biodiesel using phycocyanin from *Spirulina platensis*, the inactive biomass of *S. platensis*, and ammonium sulfate. Maximal degradation in biodiesel-contaminated soil was achieved with the addition of phycocyanin (88.75%) and in diesel-contaminated soil with the addition of inactive *S. platensis* (63.89%), confirming the advantages of using natural compounds over chemicals. Similarly, a few studies have reported the successful degradation of pesticides, such as fenanmiphos, tricyclazole, and R-endosulfan, by some species of green algae and cyanobacteria [110,111,112].

Despite the proven potential of microalgae and bacteria for the bioremediation of contaminated soils, few studies have explored their use as combined consortia. One of these studies reported that the biological degradation of high-molecular-weight PAHs was successfully achieved on long-term contaminated soil with the inoculation of the microbial consortium co-formed by the microalga *Chlorella* spp. and the bacterium *Rhodococcus wratislaviensis*. The consortium efficiently degraded phenanthrene, pyrene, and benzo[a]pyrene (BaP) to below detection levels in soil slurry within 30 days [113]. The synergy between microalgae and bacteria produces a combined effect greater than that produced by each microorganism alone. Luo et al. [114] demonstrated this when evaluating the potential of a microalga–bacterium consortium (*Selenastrum capricornutum* and *Mycobacterium* spp.) to degrade recalcitrant PAHs. In addition to a complete degradation of pyrene in 10 days, the authors reported that the bacterial degradation of pyrene mitigated its toxicity for microalgae, whose growth was substantially inhibited when growing alone. Additionally, microalgae and bacterial growth were mutually enhanced with the co-culture.

## 6. Concluding Remarks

The enormous area of degraded land worldwide demands sustainable solutions to climate change and food scarcity. However, despite the several advantages of using microbial consortia for soil restoration, there are few studies on microbial microalgae–bacteria consortia inoculants. Additionally, although the biodiversity of microalgae is huge, most studies include only filamentous cyanobacteria (mostly *Anabaena* spp.) and unicellular green microalgae (mostly *Chlorella* spp.). Furthermore, a better understanding of the synergistic interaction between cyanobacteria and bacteria is imperative for the success of restoration programs using these consortia.

Finally, most of the published information regarding the effect of microbial inoculants on soil fertility derives from studies focused on the fertilizer potential of the inoculants and their growth-promoting effect on plants; therefore, more information regarding the actual impact on degraded soils is required.

## Figures and Tables

**Table 1:** Microbial consortia of microalgae or bacteria with proven potential to enhance the characteristics of degraded soils.

Microorganisms in the Consortium	Soil Substrate	Effect on Soil Fertility	Reference
Cyanobacteria *Microcoleus vaginatus* and *Scytonema javanicum*	Desertified soils	Biocrust and moss crust formation helped with the regeneration of vascular plants.	[64]
Cyanobacteria *Microcoleus vaginatus* and *Scytonema javanicum*	Desertified soils	Significant increments in total nitrogen, organic carbon, total salt, calcium carbonate, and electrical conductivity.	[63]
Cyanobacteria *Anabaena doliolum*, *Cylindrospermum sphaerica* and *Nostoc calcicola*	Semi-arid clay–loam soil	Improved nitrogen and carbon mineralization, water-holding capacity and hydraulic conductivity.	[65]
Cyanobacteria *Microcoleus vaginatus*, *Phormidium tenue*, *Scytonema javanicum*, *Nostoc* sp. and microalga *Desmococcus olivaceus*	Aeolian sandy soil	The stabilization of fine sands and erosion control.	[66]
Microalgae *Heterochlorella* sp. MAS3 and *Desmodesmus* sp.	Acid soils	Enhanced microbial richness and diversity after 90 days of incubation.	[69]
Bacteria *Azospirillum brasilense* and *Pantoea dispersa* with organic olive residue (alperujo)	Semiarid soils	Up to 100% and 70% increments in available phosphorus and potassium content, respectively. Significant increments in total C, total organic C, and microbial biomass C content, as well as improved enzymatic activity.	[53]
Bacteria *Burkholderia* sp. RRAK1, *Pseudomonas* sp. RRAN2, *Azospirillum* sp. RRAK5, *Paenibacillus* sp. RRB2, and *Bacillus* sp. RRN12	Eroded soil	The enhancement of enzymatic activity (urease, phosphatase, β-glucosidase, phenol oxidase, dehydrogenase, and catalase).	[52]

**Table 2:** Microalgae–bacteria consortia used as inoculants with a proven ability to enhance soil fertility.

Microalgae	Bacteria	Effect on Soil Fertility	Reference
*Anabaena torulosa*	*Azotobacter chroococcum*, *Mesorhizobium ciceri*, *Serratia marcescens*, and *Pseudomonas striata*	Increased nitrogen fixing potential of up to 50% even after 14 weeks of inoculation of the biofilms.	[80]
*Anabaena oscillarioides* and *Anabaena torulosa*	*Providencia* sp. and *Alcaligenes* sp.	Enhanced dehydrogenase activity and soil microbial activity.	[82]
*Chlorella sorokiniana*	*Azospirillum brasilense*	Significant increase in soil organic matter, organic carbon, and microbial carbon.	[83]
*Chlorella sorokiniana*	*Azospirillum brasilense*	Increased soil microbial richness and diversity.	[84]
*Lyngbya* sp. *Nostoc* sp. and *Oscillatoria* sp.	*Azotobacter* sp. and *Bacillus subtilis*	Up to a 99% reduction rate in soil loss, preventing rainfall-induced soil erosion.	[85]
*Anabaena* sp.	*Providencia* sp.	Significant increment in dehydrogenase and alkaline phosphatase activity, as well as microbial biomass carbon.	[86]
*Anabaena torulosa*	*Azotobacter chroococcum*	80% increase in available N and 24% of available P over control.	[81]
*Anabaena torulosa*	*Bradyrhizobium* sp.	Significant enhancement in soil C sequestration, N_2_ fixation, and N mobilization.	[81]
*Anabaena torulosa*	*Rhizobium* sp.	Significant increase in polysaccharides, dehydrogenase and nitrogenase activity, soil carbon, and available P.	[87]
*Anabaena torulosa*	*Pseudomonas fluorescens*	Significant increase in polysaccharides, dehydrogenase and nitrogenase activity, and soil carbon.	[87]
*Anabaena torulosa*	*Mesorhizobium ciceri*	Significant increment in available soil, N and N_2_ fixation, available P, and dehydrogenase activity.	[88]

## Data Availability

Not applicable.
